# 2-Amino-4-meth­oxy-6-methyl­pyrimidin-1-ium picrate

**DOI:** 10.1107/S1600536810014583

**Published:** 2010-04-28

**Authors:** Jerry P. Jasinski, Ray J. Butcher, H. S. Yathirajan, B. Narayana, K. Prakash Kamath

**Affiliations:** a Department of Chemistry, Keene State College, 229 Main Street, Keene, NH 03435-2001, USA; b Department of Chemistry, Howard University, 525 College Street NW, Washington, DC 20059, USA; c Department of Studies in Chemistry, University of Mysore, Manasagangotri, Mysore 570 006, India; d Department of Studies in Chemistry, Mangalore University, Mangalagangotri, 574 199, India; e Department of Studies in Physics, Mangalore University, Mangalagangotri, 574 199, India

## Abstract

In the title salt, C_6_H_10_N_3_O^+^·C_6_H_2_N_3_O_7_^−^, the dihedral angle between the mean planes of the benzene and pyridine rings is 3.1 (1)°. In the cation, the meth­oxy group is almost coplanar with the pyridine ring [C—O—C—N = −0.6 (2)°]. The *p*-nitro [C—C—N—O = −1.17 (19)°] and one *o*-nitro [C—C—N—O = 1.83 (19)°] group in the anion are essentially coplanar with the benzene ring. The other disordered *o*-nitro group  containing the major occupancy [0.868 (6)] O atom is twisted −29.0 (2)° from the mean plane of the  benzene ring. A bifurcated N—H⋯(O.O) hydrogen bond and weak C—H⋯O intermolecular inter­action between the cation and anion produce a network of infinite O—H⋯O—H⋯O—H chains along the *c* axis in the [101] plane which helps to establish crystal packing. Comparison to a DFT computational calculation indicates that significant conformational changes occur in the free state.

## Related literature

For the synthesis of imidazo[1,2-*a*]pyrimidines, see: Katritzky *et al.* (2003 ▶). For related structures, see: Ferguson *et al.* (1984 ▶); Glidewell *et al.* (2003 ▶); Narayana *et al.* (2008 ▶); Scheinbeim & Schempp, (1976 ▶); Schlueter *et al.* (2006 ▶); Subashini *et al.* (2006 ▶). For density functional theory, see: Hehre *et al.* (1986 ▶); Schmidt & Polik (2007 ▶).
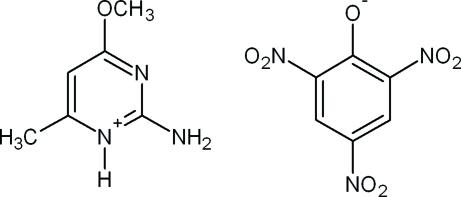


## Experimental

### 

#### Crystal data

C_6_H_10_N_3_O^+^·C_6_H_2_N_3_O_7_^−^
*M* *_r_* = 368.28
Monoclinic, 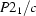

*a* = 8.9442 (3) Å
*b* = 6.2793 (3) Å
*c* = 27.0354 (8) Å
β = 94.471 (3)°
*V* = 1513.78 (10) Å^3^
*Z* = 4
Mo *K*α radiation
μ = 0.14 mm^−1^
*T* = 200 K
0.52 × 0.46 × 0.35 mm

#### Data collection

Oxford Diffraction Gemini diffractometer
Absorption correction: multi-scan (*CrysAlis RED*; Oxford Diffraction, 2007 ▶) *T*_min_ = 0.986, *T*_max_ = 1.000
15870 measured reflections
6133 independent reflections
3107 reflections with *I* > 2σ(*I*)
*R*_int_ = 0.031

#### Refinement

*R*[*F* ^2^ > 2σ(*F* ^2^)] = 0.054
*wR*(*F* ^2^) = 0.160
*S* = 0.94
6133 reflections
256 parameters
24 restraints
H-atom parameters constrained
Δρ_max_ = 0.43 e Å^−3^
Δρ_min_ = −0.29 e Å^−3^

### 

Data collection: *CrysAlis PRO* (Oxford Diffraction, 2007 ▶); cell refinement: *CrysAlis RED* (Oxford Diffraction, 2007 ▶); data reduction: *CrysAlis RED*; program(s) used to solve structure: *SHELXS97* (Sheldrick, 2008 ▶); program(s) used to refine structure: *SHELXL97*) (Sheldrick, 2008 ▶); molecular graphics: *SHELXTL* (Sheldrick, 2008 ▶); software used to prepare material for publication: *SHELXTL* and *PLATON* (Spek, 2009 ▶).

## Supplementary Material

Crystal structure: contains datablocks global, I. DOI: 10.1107/S1600536810014583/bt5240sup1.cif

Structure factors: contains datablocks I. DOI: 10.1107/S1600536810014583/bt5240Isup2.hkl

Additional supplementary materials:  crystallographic information; 3D view; checkCIF report

## Figures and Tables

**Table 1:** Hydrogen-bond geometry (Å, °)

*D*—H⋯*A*	*D*—H	H⋯*A*	*D*⋯*A*	*D*—H⋯*A*
N1*B*—H1*BA*⋯O61*B*^i^	0.88	2.09	2.883 (10)	150
N1*B*—H1*BA*⋯O61*A*^i^	0.88	2.13	2.9309 (17)	151
N1*B*—H1*BB*⋯O1*A*	0.88	1.95	2.7223 (13)	146
N1*B*—H1*BB*⋯O21*A*	0.88	2.20	2.8855 (14)	134
N2*B*—H2*BA*⋯O1*A*	0.88	1.97	2.7380 (12)	145
N2*B*—H2*BA*⋯O62*B*	0.88	2.55	3.303 (10)	144
N2*B*—H2*BA*⋯O62*A*	0.88	2.62	3.381 (2)	145

Symmetry code: (i) 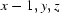.

**Fig. 1.**
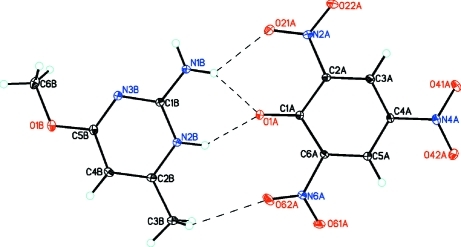
Molecular structure of the C6H10N3 O+, C6H2N3O7- cation-anion pair showing the atom labeling scheme and 50% probability displacement ellipsoids. Only the predominate component of the disordered nitro group is displayed. Dashed lines indicate hydrogen bond interactions.

**Fig. 2.**
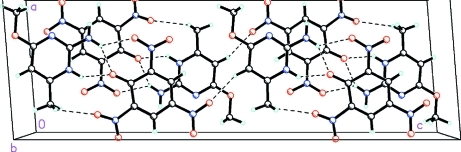
Packing diagram of the title compound, viewed down the b axis. Dashed lines indicate hydrogen bonds.

## supplementary crystallographic information

### Comment

The synthesis of imidazo[1,2-a]pyrimidines has been widely investigated and is one of the most common strategies in the use of 2-aminopyrimidine as the starting material (Katritzky *et al.*, 2003). Recently, the hydrogen-bonding patterns in 2-amino-4,6-dimethylpyrimidinium picrate has been reported (Subashini *et al.*, 2006). In continuation of our work on picrates of biologically important molecules, we have prepared a new picrate of 2-amino-4-methoxy-6-methylpyrimidine, [C_6_H_10_N_3_ O]^+^, [C_6_H_2_N_3_O_7_]^-^ and its crystal structure is reported.

The title compound,(I), C_12_H_12_N_6_O_8_, crystallizes as a salt with one C_6_H_10_N_3_ O^+^, C_6_H_2_N_3_O_7_^-^ , cation-anion pair in the asymmetric unit (Fig. 1). The dihedral angle between the mean planes of the benzene and pyridine rings is 3.10°. In the cation the methoxy group is almost coplanar with the pyridine ring [C6B-O1B-C5B-N3B torsion angle = -0.63 (19)°].  Bond distances and angles in both the cation and anion are in normal ranges (Allen, 2002). The *p* [torsion angle C3A-C4A-N4A-O41A = 1.17 (18)°] and one *o* [torsion angle C1A-C2A-N2A-O21A = 1.83 (19)°] nitro group in the anion are nearly coplanar with the benzene ring.  The other disordered *o* nitro group containing the predomoinate oxygen atom (occupancy = 0.868 (6)) is twisted -29.0 (2)\5 from the mean plane of the benzene ring.  Bifurcated intramolecular donor, N1B, [O1A [N1B—H1BB···O21A. & N1B—H1BB···O1A] and acceptor, O1A, [N1B—H1BB···O1A & N2B—H2BA···O1A] hydrogen bonds and a weak C3B—H3BB···O62A(0.868 (6)) hydrogen bond interaction (Table 2) between the cation and anion produces a network of infinite O—H···O—H···O—H chains along the *c* axis in the [101] plane which helps to establish crystal packing (Fig. 2).

A density functional theory (DFT) geometry optimization molecular orbital calculation (Schmidt & Polik, 2007) was performed on the independent cation-anion pair (C_6_H_10_N_3_ O^+^, C_6_H_2_N_3_O_7_^-^ ) within the asymmetric unit with the B3LYP 6-31-G(d) basis set (Hehre *et al.*, 1986). Starting geometries were taken from X-ray refinement data. The dihedral angle between the mean planes of the benzene and pyridine rings increases to 28.10°. In the anion, the mean planes of the two *o*-nitro groups become twisted by 23.14° and 24.20°, respectively, from the mean plane of the benzene ring. The mean plane of the *p*-nitro group remains planar to the benzene ring. The mean plane of the methoxy group in the cation also remains planar to the pyridine ring. These observations suggest that the bifurcated N—H···(O,*O*) donor and acceptor hydrogen bonds and weak C—H···O intermolecular interactions play a significant role in crystal stability.

### Experimental

4-Methoxy-6-methylpyrimidin-2-amine (1.39 g, 0.01 mol) was dissolved in 25 ml of ethanol. Picric acid (2.29 g, 0.01 mol) was dissolved in 15 ml of water. Both the solutions were mixed and to this, 5 ml of 5M HCl was added and stirred for few minutes. The formed complex was filtered and dried. Good quality crystals were grown from ethanol solution by slow evaporation (m. p.: 399 K). Composition: Found (Calculated): C: 39.09 (39.14); H: 3.24 (3.28); N: 22.77% (22.82%).

### Refinement

All of the H atoms were placed in their calculated positions and then refined using the riding model with C—H = 0.95-0.98 Å, N—H = 0.88 Å, and with U_iso_(H) = 1.18-1.52U_eq_(C,N).

### Crystal data

**Table:**

C_6_H_10_N_3_O^+^·C_6_H_2_N_3_O_7_^−^	*F*(000) = 760
*M**_r_* = 368.28	*D*_x_ = 1.616 Mg m^−^^3^
Monoclinic, *P*2_1_/*c*	Mo *K*α radiation, λ = 0.71073 Å
Hall symbol: -P 2ybc	Cell parameters from 3951 reflections
*a* = 8.9442 (3) Å	θ = 4.6–34.8°
*b* = 6.2793 (3) Å	µ = 0.14 mm^−^^1^
*c* = 27.0354 (8) Å	*T* = 200 K
β = 94.471 (3)°	Prism, yellow
*V* = 1513.78 (10) Å^3^	0.52 × 0.46 × 0.35 mm
*Z* = 4	

### Data collection

**Table:**

Oxford Diffraction Gemini diffractometer	6133 independent reflections
Radiation source: fine-focus sealed tube	3107 reflections with *I* > 2σ(*I*)
graphite	*R*_int_ = 0.031
Detector resolution: 10.5081 pixels mm^-1^	θ_max_ = 34.9°, θ_min_ = 4.6°
φ and ω scans	*h* = −11→14
Absorption correction: multi-scan (*CrysAlis RED*; Oxford Diffraction, 2007)	*k* = −9→9
*T*_min_ = 0.986, *T*_max_ = 1.000	*l* = −42→43
15870 measured reflections	

### Refinement

**Table:**

Refinement on *F*^2^	Primary atom site location: structure-invariant direct methods
Least-squares matrix: full	Secondary atom site location: difference Fourier map
*R*[*F*^2^ > 2σ(*F*^2^)] = 0.054	Hydrogen site location: inferred from neighbouring sites
*wR*(*F*^2^) = 0.160	H-atom parameters constrained
*S* = 0.94	*w* = 1/[σ^2^(*F*_o_^2^) + (0.0847*P*)^2^] where *P* = (*F*_o_^2^ + 2*F*_c_^2^)/3
6133 reflections	(Δ/σ)_max_ < 0.001
256 parameters	Δρ_max_ = 0.43 e Å^−^^3^
24 restraints	Δρ_min_ = −0.29 e Å^−^^3^

### Special details

**Table:**

Geometry. All esds (except the esd in the dihedral angle between two l.s. planes) are estimated using the full covariance matrix. The cell esds are taken into account individually in the estimation of esds in distances, angles and torsion angles; correlations between esds in cell parameters are only used when they are defined by crystal symmetry. An approximate (isotropic) treatment of cell esds is used for estimating esds involving l.s. planes.
Refinement. Refinement of *F*^2^ against ALL reflections. The weighted *R*-factor *wR* and goodness of fit *S* are based on *F*^2^, conventional *R*-factors *R* are based on *F*, with *F* set to zero for negative *F*^2^. The threshold expression of *F*^2^ > σ(*F*^2^) is used only for calculating *R*-factors(gt) *etc*. and is not relevant to the choice of reflections for refinement. *R*-factors based on *F*^2^ are statistically about twice as large as those based on *F*, and *R*- factors based on ALL data will be even larger.

### Fractional atomic coordinates and isotropic or equivalent isotropic displacement parameters (Å^2^)

**Table:**

	*x*	*y*	*z*	*U*_iso_*/*U*_eq_	Occ. (<1)
O1A	0.55766 (10)	0.25342 (17)	0.27447 (3)	0.0282 (2)	
O21A	0.30234 (11)	0.2803 (2)	0.21683 (4)	0.0407 (3)	
O22A	0.31232 (12)	0.2676 (2)	0.13799 (4)	0.0545 (4)	
O41A	0.75641 (11)	0.24825 (17)	0.05802 (3)	0.0317 (2)	
O42A	0.97162 (12)	0.2438 (2)	0.09966 (4)	0.0505 (4)	
O61A	1.00309 (15)	0.1699 (4)	0.27556 (5)	0.0322 (5)	0.868 (6)
O62A	0.83577 (17)	0.3370 (5)	0.31386 (5)	0.0433 (6)	0.868 (6)
O61B	1.0068 (10)	0.311 (3)	0.2751 (3)	0.040 (3)	0.132 (6)
O62B	0.8309 (11)	0.195 (3)	0.3157 (3)	0.038 (3)	0.132 (6)
O1B	0.28372 (11)	0.24739 (17)	0.49248 (3)	0.0326 (2)	
N2A	0.37356 (11)	0.26925 (19)	0.18027 (4)	0.0241 (2)	
N4A	0.83482 (12)	0.24677 (19)	0.09742 (4)	0.0251 (2)	
N6A	0.87761 (11)	0.25092 (18)	0.27698 (4)	0.0222 (2)	
N1B	0.30287 (11)	0.25263 (17)	0.32338 (3)	0.0193 (2)	
H1BA	0.2044	0.2490	0.3191	0.023*	
H1BB	0.3568	0.2555	0.2975	0.023*	
N2B	0.52320 (11)	0.25912 (18)	0.37421 (3)	0.0194 (2)	
H2BA	0.5741	0.2630	0.3477	0.023*	
N3B	0.28659 (11)	0.24940 (18)	0.40787 (3)	0.0209 (2)	
C1A	0.61812 (12)	0.2561 (2)	0.23423 (4)	0.0173 (2)	
C2A	0.53717 (12)	0.2594 (2)	0.18562 (4)	0.0175 (2)	
C3A	0.60696 (13)	0.2561 (2)	0.14188 (4)	0.0183 (2)	
H3AA	0.5490	0.2588	0.1109	0.022*	
C4A	0.76183 (13)	0.2487 (2)	0.14336 (4)	0.0184 (2)	
C5A	0.85022 (12)	0.2451 (2)	0.18818 (4)	0.0186 (2)	
H5AA	0.9565	0.2389	0.1888	0.022*	
C6A	0.77977 (12)	0.2506 (2)	0.23132 (4)	0.0173 (2)	
C1B	0.36982 (12)	0.2538 (2)	0.36869 (4)	0.0169 (2)	
C2B	0.59876 (14)	0.2586 (2)	0.42009 (4)	0.0243 (3)	
C3B	0.76596 (15)	0.2631 (3)	0.42302 (5)	0.0375 (4)	
H3BA	0.8052	0.2642	0.4579	0.056*	
H3BB	0.7996	0.3914	0.4065	0.056*	
H3BC	0.8030	0.1366	0.4066	0.056*	
C4B	0.51754 (14)	0.2534 (3)	0.46052 (5)	0.0300 (3)	
H4BA	0.5652	0.2521	0.4932	0.036*	
C5B	0.35982 (14)	0.2501 (2)	0.45220 (4)	0.0235 (3)	
C6B	0.12228 (16)	0.2460 (3)	0.48535 (5)	0.0344 (3)	
H6BA	0.0799	0.2454	0.5177	0.052*	
H6BB	0.0891	0.1184	0.4667	0.052*	
H6BC	0.0880	0.3732	0.4668	0.052*	

### Atomic displacement parameters (Å^2^)

**Table:**

	*U*^11^	*U*^22^	*U*^33^	*U*^12^	*U*^13^	*U*^23^
O1A	0.0188 (4)	0.0497 (7)	0.0167 (4)	0.0001 (4)	0.0047 (3)	0.0008 (4)
O21A	0.0198 (4)	0.0763 (9)	0.0263 (5)	0.0020 (5)	0.0042 (4)	−0.0057 (5)
O22A	0.0213 (5)	0.1159 (13)	0.0248 (5)	−0.0006 (6)	−0.0071 (4)	0.0054 (6)
O41A	0.0354 (5)	0.0456 (6)	0.0138 (4)	−0.0003 (5)	−0.0010 (3)	0.0000 (4)
O42A	0.0219 (5)	0.1061 (12)	0.0246 (5)	0.0017 (6)	0.0091 (4)	0.0009 (6)
O61A	0.0179 (6)	0.0502 (13)	0.0273 (6)	0.0077 (7)	−0.0054 (4)	−0.0034 (7)
O62A	0.0343 (7)	0.0769 (18)	0.0180 (6)	0.0094 (9)	−0.0035 (5)	−0.0166 (8)
O61B	0.021 (4)	0.077 (9)	0.023 (4)	−0.006 (5)	−0.005 (3)	0.002 (5)
O62B	0.023 (4)	0.069 (8)	0.022 (4)	−0.009 (5)	0.001 (3)	0.008 (4)
O1B	0.0272 (5)	0.0534 (7)	0.0183 (4)	−0.0004 (5)	0.0076 (3)	0.0000 (4)
N2A	0.0153 (4)	0.0341 (7)	0.0226 (5)	−0.0017 (5)	−0.0001 (4)	0.0013 (5)
N4A	0.0252 (5)	0.0352 (6)	0.0153 (4)	−0.0001 (5)	0.0031 (4)	0.0001 (5)
N6A	0.0180 (4)	0.0324 (6)	0.0157 (4)	−0.0022 (5)	−0.0016 (3)	0.0014 (5)
N1B	0.0148 (4)	0.0265 (5)	0.0163 (4)	0.0003 (5)	−0.0002 (3)	−0.0010 (4)
N2B	0.0152 (4)	0.0278 (6)	0.0152 (4)	−0.0004 (5)	0.0001 (3)	0.0002 (4)
N3B	0.0180 (4)	0.0273 (6)	0.0177 (4)	0.0012 (5)	0.0025 (3)	0.0001 (4)
C1A	0.0162 (5)	0.0202 (6)	0.0155 (4)	0.0003 (5)	0.0019 (4)	−0.0006 (5)
C2A	0.0143 (4)	0.0208 (6)	0.0171 (5)	−0.0007 (5)	−0.0005 (4)	−0.0006 (5)
C3A	0.0196 (5)	0.0191 (6)	0.0158 (4)	−0.0012 (5)	−0.0011 (4)	0.0003 (5)
C4A	0.0196 (5)	0.0229 (6)	0.0129 (4)	0.0003 (5)	0.0031 (4)	0.0006 (5)
C5A	0.0159 (5)	0.0229 (6)	0.0168 (5)	−0.0005 (5)	0.0007 (4)	0.0007 (5)
C6A	0.0163 (5)	0.0218 (6)	0.0134 (4)	0.0010 (5)	−0.0014 (3)	0.0004 (5)
C1B	0.0154 (5)	0.0172 (5)	0.0181 (5)	0.0001 (5)	0.0008 (4)	−0.0002 (5)
C2B	0.0170 (5)	0.0354 (7)	0.0200 (5)	−0.0001 (6)	−0.0023 (4)	0.0009 (5)
C3B	0.0174 (5)	0.0706 (12)	0.0235 (6)	−0.0024 (7)	−0.0037 (4)	0.0003 (7)
C4B	0.0225 (6)	0.0505 (9)	0.0164 (5)	−0.0006 (7)	−0.0022 (4)	−0.0003 (6)
C5B	0.0227 (5)	0.0320 (7)	0.0162 (5)	−0.0005 (6)	0.0039 (4)	0.0009 (5)
C6B	0.0252 (6)	0.0494 (9)	0.0302 (6)	−0.0014 (7)	0.0124 (5)	−0.0013 (7)

### Geometric parameters (Å, °)

**Table:**

O1A—C1A	1.2523 (13)	N3B—C5B	1.3204 (15)
O21A—N2A	1.2190 (13)	N3B—C1B	1.3416 (14)
O22A—N2A	1.2284 (14)	C1A—C2A	1.4503 (15)
O41A—N4A	1.2287 (13)	C1A—C6A	1.4545 (16)
O42A—N4A	1.2205 (14)	C2A—C3A	1.3797 (15)
O61A—N6A	1.2357 (17)	C3A—C4A	1.3834 (16)
O62A—N6A	1.2182 (17)	C3A—H3AA	0.9500
O61B—N6A	1.221 (10)	C4A—C5A	1.3944 (15)
O62B—N6A	1.210 (9)	C5A—C6A	1.3685 (15)
O1B—C5B	1.3283 (14)	C5A—H5AA	0.9500
O1B—C6B	1.4418 (17)	C2B—C4B	1.3593 (17)
N2A—C2A	1.4605 (14)	C2B—C3B	1.4916 (18)
N4A—C4A	1.4477 (14)	C3B—H3BA	0.9800
N6A—C6A	1.4565 (14)	C3B—H3BB	0.9800
N1B—C1B	1.3210 (14)	C3B—H3BC	0.9800
N1B—H1BA	0.8800	C4B—C5B	1.4112 (18)
N1B—H1BB	0.8800	C4B—H4BA	0.9500
N2B—C2B	1.3654 (15)	C6B—H6BA	0.9800
N2B—C1B	1.3687 (14)	C6B—H6BB	0.9800
N2B—H2BA	0.8800	C6B—H6BC	0.9800
			
C5B—O1B—C6B	117.54 (10)	C3A—C4A—C5A	121.63 (10)
O21A—N2A—O22A	122.12 (11)	C3A—C4A—N4A	119.53 (10)
O21A—N2A—C2A	120.33 (10)	C5A—C4A—N4A	118.84 (10)
O22A—N2A—C2A	117.55 (10)	C6A—C5A—C4A	118.20 (10)
O42A—N4A—O41A	123.06 (10)	C6A—C5A—H5AA	120.9
O42A—N4A—C4A	118.35 (10)	C4A—C5A—H5AA	120.9
O41A—N4A—C4A	118.59 (10)	C5A—C6A—C1A	124.91 (10)
O62B—N6A—O62A	43.1 (8)	C5A—C6A—N6A	115.86 (10)
O62B—N6A—O61B	121.1 (6)	C1A—C6A—N6A	119.22 (9)
O62A—N6A—O61B	104.4 (6)	N1B—C1B—N3B	119.52 (10)
O62B—N6A—O61A	106.5 (6)	N1B—C1B—N2B	118.64 (9)
O62A—N6A—O61A	123.08 (13)	N3B—C1B—N2B	121.84 (10)
O61B—N6A—O61A	42.4 (9)	C4B—C2B—N2B	118.22 (11)
O62B—N6A—C6A	120.6 (5)	C4B—C2B—C3B	123.65 (11)
O62A—N6A—C6A	119.45 (12)	N2B—C2B—C3B	118.13 (11)
O61B—N6A—C6A	118.3 (4)	C2B—C3B—H3BA	109.5
O61A—N6A—C6A	117.40 (10)	C2B—C3B—H3BB	109.5
C1B—N1B—H1BA	120.0	H3BA—C3B—H3BB	109.5
C1B—N1B—H1BB	120.0	C2B—C3B—H3BC	109.5
H1BA—N1B—H1BB	120.0	H3BA—C3B—H3BC	109.5
C2B—N2B—C1B	121.33 (9)	H3BB—C3B—H3BC	109.5
C2B—N2B—H2BA	119.3	C2B—C4B—C5B	117.54 (11)
C1B—N2B—H2BA	119.3	C2B—C4B—H4BA	121.2
C5B—N3B—C1B	116.75 (10)	C5B—C4B—H4BA	121.2
O1A—C1A—C2A	124.65 (10)	N3B—C5B—O1B	119.64 (11)
O1A—C1A—C6A	123.03 (10)	N3B—C5B—C4B	124.31 (10)
C2A—C1A—C6A	112.30 (9)	O1B—C5B—C4B	116.05 (11)
C3A—C2A—C1A	123.31 (10)	O1B—C6B—H6BA	109.5
C3A—C2A—N2A	115.63 (10)	O1B—C6B—H6BB	109.5
C1A—C2A—N2A	121.06 (9)	H6BA—C6B—H6BB	109.5
C2A—C3A—C4A	119.64 (10)	O1B—C6B—H6BC	109.5
C2A—C3A—H3AA	120.2	H6BA—C6B—H6BC	109.5
C4A—C3A—H3AA	120.2	H6BB—C6B—H6BC	109.5
			
O1A—C1A—C2A—C3A	−178.06 (12)	C2A—C1A—C6A—N6A	178.84 (11)
C6A—C1A—C2A—C3A	0.43 (19)	O62B—N6A—C6A—C5A	−158.8 (10)
O1A—C1A—C2A—N2A	2.7 (2)	O62A—N6A—C6A—C5A	151.0 (2)
C6A—C1A—C2A—N2A	−178.78 (11)	O61B—N6A—C6A—C5A	22.2 (11)
O21A—N2A—C2A—C3A	−177.43 (13)	O61A—N6A—C6A—C5A	−26.1 (2)
O22A—N2A—C2A—C3A	2.02 (18)	O62B—N6A—C6A—C1A	21.3 (10)
O21A—N2A—C2A—C1A	1.83 (19)	O62A—N6A—C6A—C1A	−29.0 (2)
O22A—N2A—C2A—C1A	−178.72 (13)	O61B—N6A—C6A—C1A	−157.8 (11)
C1A—C2A—C3A—C4A	0.2 (2)	O61A—N6A—C6A—C1A	153.92 (16)
N2A—C2A—C3A—C4A	179.42 (12)	C5B—N3B—C1B—N1B	179.78 (12)
C2A—C3A—C4A—C5A	−0.2 (2)	C5B—N3B—C1B—N2B	−0.12 (19)
C2A—C3A—C4A—N4A	−179.57 (12)	C2B—N2B—C1B—N1B	−179.40 (12)
O42A—N4A—C4A—C3A	178.82 (13)	C2B—N2B—C1B—N3B	0.5 (2)
O41A—N4A—C4A—C3A	−1.17 (19)	C1B—N2B—C2B—C4B	−0.2 (2)
O42A—N4A—C4A—C5A	−0.59 (19)	C1B—N2B—C2B—C3B	179.57 (13)
O41A—N4A—C4A—C5A	179.42 (13)	N2B—C2B—C4B—C5B	−0.3 (2)
C3A—C4A—C5A—C6A	−0.5 (2)	C3B—C2B—C4B—C5B	179.86 (15)
N4A—C4A—C5A—C6A	178.91 (12)	C1B—N3B—C5B—O1B	179.54 (12)
C4A—C5A—C6A—C1A	1.2 (2)	C1B—N3B—C5B—C4B	−0.5 (2)
C4A—C5A—C6A—N6A	−178.78 (12)	C6B—O1B—C5B—N3B	−0.6 (2)
O1A—C1A—C6A—C5A	177.37 (13)	C6B—O1B—C5B—C4B	179.43 (14)
C2A—C1A—C6A—C5A	−1.14 (19)	C2B—C4B—C5B—N3B	0.8 (2)
O1A—C1A—C6A—N6A	−2.6 (2)	C2B—C4B—C5B—O1B	−179.30 (14)

### Hydrogen-bond geometry (Å, °)

**Table:**

*D*—H···*A*	*D*—H	H···*A*	*D*···*A*	*D*—H···*A*
N1B—H1BA···O61B^i^	0.88	2.09	2.883 (10)	150
N1B—H1BA···O61A^i^	0.88	2.13	2.9309 (17)	151
N1B—H1BB···O1A	0.88	1.95	2.7223 (13)	146
N1B—H1BB···O21A	0.88	2.20	2.8855 (14)	134
N2B—H2BA···O1A	0.88	1.97	2.7380 (12)	145
N2B—H2BA···O62B	0.88	2.55	3.303 (10)	144
N2B—H2BA···O62A	0.88	2.62	3.381 (2)	145

Symmetry codes: (i) *x*−1, *y*, *z*.
